# The effects of lisdexamfetamine dimesylate on eating behaviour and homeostatic, reward and cognitive processes in women with binge-eating symptoms: an experimental medicine study

**DOI:** 10.1038/s41398-021-01770-4

**Published:** 2022-01-10

**Authors:** Elizabeth Schneider, Elizabeth Martin, Pia Rotshtein, Kasim L. Qureshi, Samuel R. Chamberlain, Maartje S. Spetter, Colin T. Dourish, Suzanne Higgs

**Affiliations:** 1 School of Psychology, University of Birmingham, Edgbaston, Birmingham, B15 2TT United Kingdom; 2 Centre for Human Brain Health, University of Birmingham, Birmingham, United Kingdom; 3 Department of Psychiatry, Faculty of Medicine, University of Southampton, Southampton, United Kingdom; 4 Southern Health NHS Foundation Trust, Southampton, United Kingdom; 5 P1vital Ltd, Howbery Park, Wallingford, United Kingdom; 6 P1vital Products Ltd, Howbery Park, Wallingford, United Kingdom

**Keywords:** Clinical pharmacology, Human behaviour

## Abstract

Lisdexamfetamine dimesylate (LDX) is the only drug currently approved by the FDA for the treatment of Binge-Eating Disorder (BED), but little is known about the behavioural mechanisms that underpin the efficacy of LDX in treating BED. We examined the behavioural and neural effects of an acute dose of LDX (50 mg) in 22 women with binge-eating symptomatology using a randomised, crossover, double-blind, placebo-controlled experimental medicine design. LDX reduced self-reported appetite ratings and intake of both a pasta meal and a palatable cookie snack. LDX also decreased the eating rate of pasta but not of cookies and reduced self-reported liking ratings for pasta at the end of the meal. When viewing food pictures during an fMRI scan, LDX reduced activity bilaterally in the thalamus. LDX enhanced sustained attention and reduced impulsive responding in a continuous performance task but had no effect on emotional bias or working memory. These results suggest the observed effects of LDX on food intake (and by implication the efficacy of LDX in treating BED) may be related to the actions of the drug to enhance satiety, reduce food-related reward responding when full and/or increase cognitive control. Novel pharmacotherapies for BED might be most effective if they have a broad spectrum of effects on appetite, reward and cognition.

## Introduction

Binge-eating disorder (BED) is the most common specific eating disorder, and the estimated lifetime global prevalence is between 0.9–2.2.% [1]. In 2015, the United States Food and Drug Administration (FDA) approved lisdexamfetamine dimesylate (LDX) (Vyvanse^®^, Takeda) as the first and, to date, only drug for the treatment of BED [2]. This approval was based on the results of two phase-III, 12-week randomised, double-blind, multi-centre, parallel-group, placebo-controlled, dose-optimisation studies in adults with BED [2]. In both studies, 50 and 70 mg LDX reduced binge-eating episodes and weight as compared to placebo [3, 4]. Since then, evidence has accumulated to suggest that LDX is an effective treatment for BED [5, 6]. There is also evidence that LDX reduces food intake in preclinical models of binge eating in rodents [7, 8].

A recent systematic review and meta-analysis of both clinical and preclinical studies on the effects of LDX on binge eating found a poverty of studies examining the neural and cognitive processes that underpin LDX’s effects in ameliorating BED symptoms [9]. Schneider et al. [9] proposed that LDX may reduce binge eating by a combination of effects on appetite/satiety, reward, and cognitive processes that are mediated by actions of LDX on catecholaminergic and serotoninergic transmission. There is considerable potential to use the power of experimental medicine to explore the mechanism of action of LDX in treating BED. Only a single pilot fMRI study with LDX in BED has been conducted to date [10], and this study did not include a placebo control group. LDX is a stimulant drug and classified as a Schedule II controlled substance by the US Drug Enforcement Administration (DEA). An improved understanding of the neuropsychological processes impacted by LDX could aid in the development of novel medications to treat BED, with improved efficacy and fewer side effects that are not schedule controlled by the DEA.

Accordingly, we conducted a multimodal study to investigate the behavioural and neural mechanisms that underlie the effects of LDX on binge eating. We enroled participants with above threshold scores on a self-reported measure of binge-eating symptomatology. This approach is in line with the Research Domain Criteria Initiative (RDoC) established by the US National Institute of Mental Health (NIMH), which encourages research on dimensions of observable behaviour rather than a diagnostic approach to the study of mental health symptoms [11]. Binge-like eating was modelled using a paradigm in which participants first consumed a pasta meal to the point of satiety and were then offered palatable cookies to consume *ad libitum* [12]. Satiety (homeostatic) and reward processes were assessed by examining specific components of eating behaviour [13, 14]. Previous studies have established that an increase in satiety is reflected by a decrease in eating rate, whereas reduced reward is reflected in decreased palatability responses at the start of a meal [15, 16]. Inhibitory control (relevant to impulsivity) was assessed using the stop-signal task [17]. Attentional processing was assessed using a continuous performance task, and working memory was indexed by performance on an n-back task [18]. To examine effects on mood, participants rated their mood throughout the study using visual analogue scales (VAS) and completed tests of emotional processing from the P1vital^®^ Oxford Emotional Test Battery (ETB) [19–21]. The underpinning neural mechanisms were examined using task-based fMRI. We hypothesised that participants would consume less pasta and cookies in the LDX condition than the placebo condition and that eating rate and palatability ratings would also decrease in the LDX condition compared to the placebo condition. We further hypothesised that LDX would improve performance on cognitive tasks versus placebo. Finally, we predicted that LDX might reduce neural responses to the viewing of food pictures in areas of the brain involved in reward and homeostatic processes.

## Materials and methods

### Participants

Twenty-three women with binge eating were recruited for the study. One participant withdrew from the study due to vomiting during the test day. Unblinding revealed the participant had received LDX on this test day. The sample size was based on the results of a previous study that assessed the effects of a 5-HT_2C_ receptor agonist on food intake using similar paradigms (effect size of 0.67) [12]. A power analysis (G*power 3.1.9.7) [22] indicated a sample size of 20 participants was needed to obtain 80% power to detect such an effect at alpha = 0.05. To allow for smaller effect size and for dropouts, we initially aimed to recruit 35 participants. However, due to the global pandemic caused by the novel coronavirus SARS-CoV-2, all in-person data collection was halted, and the resulting sample size was 22 (*M* age = 24.41 ± 6.87, *M* BMI = 26.35 ± 4.98).

Participants were invited to take part if they met the eligibility criteria (See Supplementary Table 1) and were recruited via posters and social media platforms. The study was approved by the National Research Ethics Service and was pre-registered on clinicaltrials.gov as NCT04181957.

### Design

In a double-blind, placebo-controlled, crossover design, participants meeting inclusion criteria, and having given full informed consent, were randomised prior to the test day by a researcher not involved in data collection to receive oral LDX (50 mg) in a single morning dose, or placebo, in a counterbalanced order. The LDX and placebo were prepared by Guy’s and St Thomas’ NHS Foundation Trust Pharmacy. Both LDX and placebo were prepared in identical capsules to maintain blinding. Previous research indicates that 50 mg LDX is a clinically effective dose with few side effects [3, 4]. All participants took part in two sessions on two separate days, at least 7 days apart.

### Eating-related measures

Food was served on the Sussex Ingestion Pattern Monitor (SIPM), which consists of a balance placed underneath the surface of a table covered by a placemat [12]. The balance was connected to a laptop that recorded the weight of the plate and alerted the participant each time 50 mg of pasta was consumed, or 10 g of cookies was consumed, at which point the participant was instructed to complete VAS ratings of hunger, fullness, and pleasantness of the meal. Eating rate was calculated as grams eaten/total time spent eating (minutes). Lunch comprised pasta shells in a tomato and herb sauce (both Sainsbury’s brand) served at 55–60 °C (233 kilocalories per 200 g) and *ad libitum* water. After 150 g had been consumed, the participants were interrupted, and the plate was replaced with a fresh 200 g plate of pasta. Participants were instructed to continue to eat as many plates as they wished until they were comfortably full. Maryland brand chocolate chip cookies were offered *ad libitum* 15 min after the pasta meal. Participants were served a bowl containing 80 g (approximately 396 kilocalories) of cookies broken into bite-size amounts to avoid participant tracking of amount consumed. When 60 g of cookies were consumed, participants were provided with a fresh bowl containing 80 g and could continue in this manner until they wished to stop.

### Cognitive Tasks

#### P1vital^®^ Oxford emotional test battery (ETB)

The ETB is a computerised battery that comprises validated cognitive tasks to determine emotional bias [20].

*Emotional categorisation (ECAT)*: Sixty positive and negative adjectives (e.g., cheerful, hostile) were presented in white text on a black screen. Each adjective was presented for 500 ms. The participant was instructed to select if they would ‘like’ or ‘dislike’ to be described as such as quickly and accurately as possible. Accuracy and reaction time (RT) by valence are reported.

*Emotional recall (EREC)*: The participants were asked to recall as many words from the ECAT as could be remembered within a 4 min period. Participants wrote their responses on paper. The number of correct words recalled by valence and commission errors are reported.

*Emotional recognition memory (EMEM)*: Participants were presented with the 60 words from the ECAT, along with 60 matching novel distractor words, on a black screen. The participants were instructed to indicate whether the word had been presented during the ECAT trial. Accuracy, RT, and commission errors by valence are reported.

*Facial expression recognition (FERT):* Faces with one of six emotional expressions (happiness, fear, anger, disgust, sadness and surprise) or a neutral expression appeared on a black background screen. The faces were morphed from neutral to full expressions in 10% increments to foster ambiguity about the expression being displayed. Each intensity was represented four times, along with ten presentations of neutral expressions totalling 250 stimuli. Each stimulus was presented for 500 ms, followed by a blank screen. The participant was instructed to classify each expression as quickly and as accurately as possible. Accuracy, commission errors, and RT by valence are reported.

#### Stop-signal task (SST)

The SST is a measure of response inhibition [23]. This task was adapted from the STOP-IT software programmed by Verbruggen et al. [23]. A white arrow was presented on a black background, pointing either left or right. The participant indicated the direction of the arrow using the left and right keys on the keyboard. On a subset of these trials (‘stop trials’), the white arrow turned blue in colour, indicating that the participant had to attempt to inhibit their motor response on the given trial (as instructed in advance of doing the paradigm). The blue arrow in stop-signal trials is initially presented for 250 ms, and this delay is then adjusted using the staircase tracking procedure whereby the personalised adjusted score is the stop-signal delay (SSD). The experiment consists of three blocks of 64 trials in which 75% of the trials are no-signal trials. The stop-signal reaction times (SSRT) indicates the time taken by the individual to suppress a response that would normally be made and is calculated by subtracting mean SSD from mean RT. Omission and commission errors, RT for no-signal and stop-signal trials (SSRT), and SSD are reported.

#### N-back

The participant was presented with a sequence of blue circles on a white 3 × 3 grid and was instructed to indicate whether the current circle location matched or did not match the location of the circle 2 (2-back) or 3 (3-back) trials earlier. Participants completed 70 trials of each condition with a break between the 2 and 3-back. Accuracy and RT for each condition (2 back or 3 back) are reported.

#### Continuous performance test

A series of white letters were presented on a grey background in a random order [24, 25]. Participants were instructed to press the space bar for every letter except ‘X’. Letters were presented for 900 ms. The ‘X’ appeared in 42 of the 830 trials. An average of the RT standard deviations (SDRT) was calculated to measure response time variability (RTV). Increased RTV is considered to reflect poorer ability to sustain attention [26]. Commission errors provide a measure of impulsive responding, while omission errors provide a measure of inattention [27]. Omission and commission errors, RT and SDRT/RTV are reported.

### fMRI picture rating task

During fMRI, participants performed a picture rating task [12]. Participants viewed food and non-food stimuli (36 from each category and visually matched). The food pictures varied in fat and sugar content (high fat, high sugar; high fat, low sugar; low fat, high sugar and low fat, low sugar). Items were rated on how appealing they were on a scale from 1 (not at all) to 5 (very much) using a button box. Each picture was presented for 1500 ms, followed by a fixation cross (500–1500 ms).

### Acquisition, processing and analysis of fMRI data

Imaging data were collected using a Siemens MAGNETOM Prisma 3 T MRI system at the Centre for Human Brain Health (CHBH), University of Birmingham. Functional images during the picture rating task (3× 300 volumes) were acquired with single-shot echo-planar imaging (EPI) sequence as described in the supplementary materials. Data were analysed using SPM12 (Wellcome Department of Imaging Neuroscience, London, UK) run with MATLAB 2019 (Mathworks Inc, Natick, MA) using standard procedures [28] (supplementary materials).

### Procedure

**Fig. 1:**
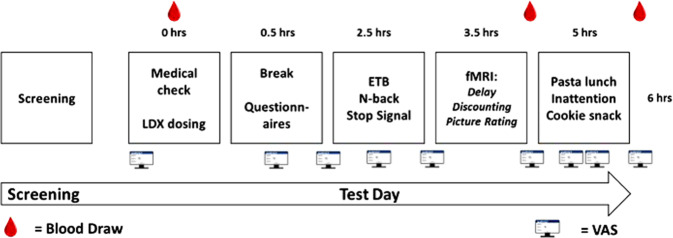
Timeline of screening and testing. Flow diagram showing an overview of the screening and test days timings in hours (hrs).

Participants attended a screening day and a separate test day. On the screening day, binge eating was confirmed using the Binge-Eating Scale (BES) [29]. Participants were eligible to take part if they had a Moderate score (18–26) or Severe score (27–46). The Structured Clinical Interview for DSM-5, Clinical Version (SCID-CV) [30] was also completed on the screening day to exclude participants with other mental health conditions, including Anorexia Nervosa and Bulimia Nervosa. On the test day, participants arrived at 8:30 or 9:00 after eating their usual breakfast (to standardise hunger). After the LDX or placebo capsule was self-administered, participants waited for 2 h for peak drug levels to be achieved and during this time they completed the Dutch Eating Behaviour Questionnaire (DEBQ) [31], and the Barratt Impulsiveness Scale (BIS-11) [32]. From 11:00 am they completed the following tasks in order: ETB, SST, n-back, fMRI session. The fMRI session started around 12:30. During the fMRI scan, participants completed a delay discounting task (data not reported here) and the picture rating task. Following the scan, lunch was offered and then participants completed the inattention task before consuming the cookie snack. Throughout the day, VAS assessing mood and physical state were completed. A total of 14 items were rated using a 0 cm (‘completely absent’) to 10 cm (‘most I could imagine’) scale: alertness, drowsiness, happiness, hunger, fullness, desire to eat, disgust, anxiety, sadness, withdrawn, lightheaded, nausea and faint. A total of 3 mL blood samples were collected via venipuncture for assessment of d-amphetamine concentration (mg/L) by Analytical Services International Ltd. The results confirmed no presence of d-amphetamine in baseline blood samples and expected plasma levels of D-amphetamine after 275 min (0.05 mg/L) and 325 min (0.06 mg/L) post-dosing (see Fig. 1 for a summary of the test day).

### Data and statistical analysis

Performance-based exclusion criteria were determined prior to data analysis. Outlying data points, which were defined as below 200 ms and ≥ 6000 ms for RTs and outside 3*interquartile range of the lower and upper grand mean for other performance measures were removed. In addition, participants scoring at below chance performance (less than 50%) on the EMEM and N-back tasks were removed from the analysis, which resulted in smaller degrees of freedom for these tasks. Using the factor structure calculated by Thomas et al. [15], VAS factors consisted of ‘Arousal’ (alertness, drowsiness, and happiness), ‘Appetite’ (hunger, fullness, and desire to eat), ‘Negative Effects’ (disgust, anxiety, sadness, and withdrawn), ‘Physical Effects’ (lightheaded, nausea, and faint), and thirst [15]. VAS factors were converted to AUC using the trapezoid method. Regression imputations were used to replace missing VAS data. The data met the assumptions for parametric testing. Unless noted otherwise, the data were analysed using repeated-measures ANOVA. Main effects and interactions that did not involve drug conditions are not reported and comparisons are reported for LDX versus placebo.

## Results

### Participant characteristics

The women were on average overweight (mean BMI = 26.35 (4.98)), and the majority (59%) scored Severe on the BES (mean score 28.36 (6.59)) (see Supplemental Table 2 for scores on the DEBQ, BDI, BIS).

### Food intake eating rate and food liking

**Fig. 2:**
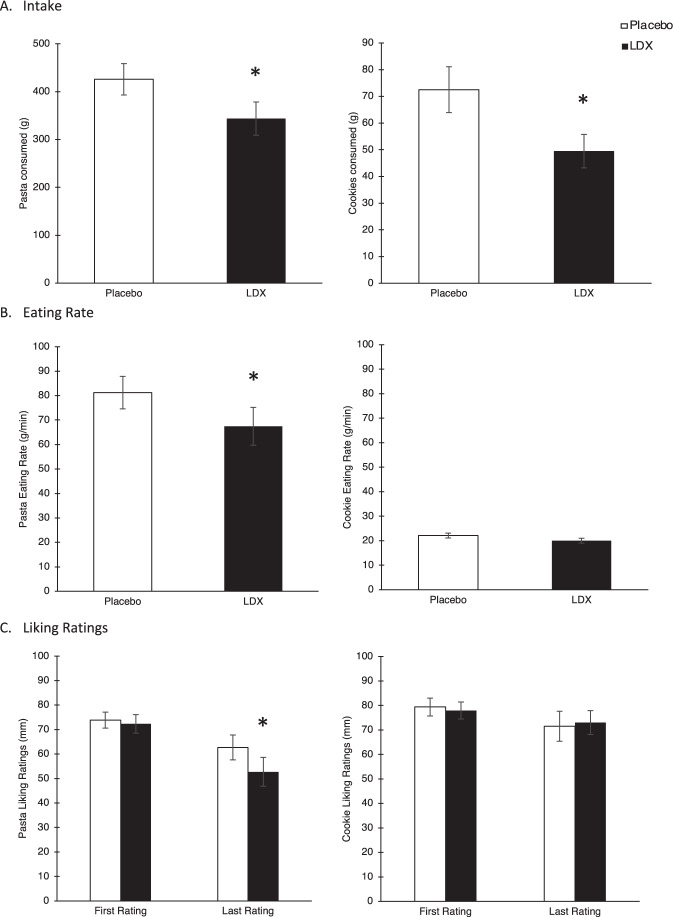
Eating-related measures. **A** Intake of pasta and cookies for the placebo (open bars) and LDX (filled bars) conditions. **B** Eating rate of pasta and cookies for the placebo (open bars) and LDX (filled bars) conditions. **C** Liking ratings of pasta and cookies for the placebo (open bars) and LDX (filled bars) conditions. Error bars represent standard error of the mean. Asterisk denotes significantly different from placebo (*p* < 0.05).

The interaction between drug condition and food type was significant (*F*(1, 21) = 4.42, *p* = 0.048, η_p_^2^ = 0.17). Follow-up *t-*tests showed that LDX reduced intake of both pasta (*t*(21) = −2.83, *p* = 0.01, *d* = 0.52) and cookies (*t*(21) = −4.284, *p* < 0.01, *d* = 0.65), but the effect size was larger for cookies than for pasta. For eating rate, the interaction between drug condition and food type was significant (*F*(1, 20) = 5.80, *p* = 0.03, η_p_^2^ = 0.23). Follow-up tests indicated participants had a reduced eating rate after LDX versus placebo for pasta (*t*(21) = −3.14, *p* = 0.01, *d* = 0.46) but not for cookies (*t*(20) = −1.54, *p* = 0.14, *d* = 0.23). For liking ratings, the interaction between drug condition, food type, and time approached significance (*F*(1, 21) = 4.24, *p* = 0.05, η_p_^2^ = 0.17) such that pasta was rated as less liked at the end of the meal after LDX versus placebo (*t*(21) = −2.57, *p* = 0.018) but not at the start of the meal (Fig. 2).

### Mood

**Fig. 3:**
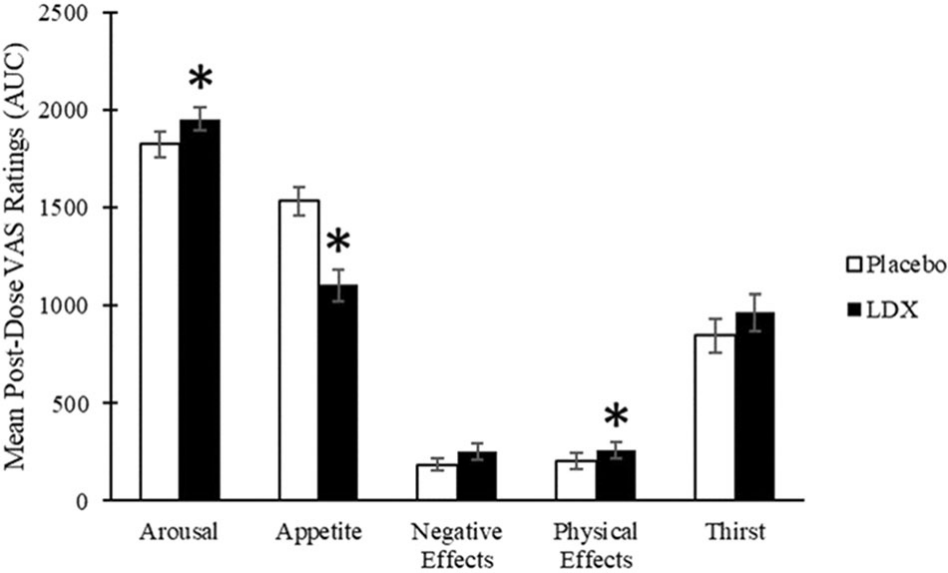
Visual analogue scales (VAS) ratings presented as means for area under the curve (AUC) for the placebo (open bars) and LDX (filled bars) conditions. Error bars represent the standard error of the mean. Asterisks denote significantly different from placebo (*p* < 0 .05).

There were no baseline differences in mood. LDX increased post-dose ratings of arousal (*t*(21) = 3.11, *p* = 0.01, *d* = 0.46) and physical effects (*t*(21) = 3.11, *p* = 0.01, *d* = 0.28) and reduced appetite (*t*(21) = −6.62, *p* < 0.01, *d* = 1.18) relative to placebo. LDX had no effect on thirst (*t*(21) = 1.41, *p* = 0.17, *d* = 0.27) and the effect of LDX to increase negative effects approached significance (*t*(21) = 2.07, *p* = 0.05, *d* = 0.38) (Fig. 3).

### Cognition

#### SST

The effect of LDX on stop-signal commission errors was marginally significant: LDX reduced commission errors (*t*(20) = −1.97, *p* = 0.06, *d* = 0.43), but there was no effect on omission errors (*t*(19) = 0.67, *p* = 0.51, *d* = 0.15), no-signal RT (*t*(20) = 1.59, *p* = 0.13, *d* = 0.35), SSD (*t*(20) = 1.20 *p* = 0.24, *d* = 0.26), nor SSRT (*t*(19) = −0.15, *p* = 0.88, *d* = −0.03) (Table 1).

**Table 1:** Stop-signal task (SST) and continuous performance task (CPT) results.

Measure	Placebo ( ± SE)	LDX ( ± SE)
SST		
No-signal omission error (%)	5.66 (1.50)	6.58 (1.24)
No-signal reaction time (ms)	919.66 (57.60)	989.26 (55.81)
Stop-signal commission error (%)	47.11 (0.59)	45.88 (0.55)
Stop-signal reaction time (SSRT; ms)	194.71 (7.06)	192.33 (13.46)
Stop-signal delay (SSD; ms)	723.56 (62.84)	786.31 (59.07)
CPT		
Target omission errors (%)	3.75 (0.34)	3.58 (0.16)
Target reaction time (ms)	340.91 (9.81)	349.32 (8.10)
Non-target commission errors (%)	39.29 (3.88)	31.31 (3.21)*
SDRT/RTV (ms)	123.03 (6.38)	108/09 (6.33)*

Results presented as means and standard error of the mean. *SDRT* Standard deviation of the reaction times, *RTV* Response time variability. *LDX significantly (*p* < 0.05) reduced non-target commission errors and SDRT/RTV compared to placebo for the CPT.

#### Continuous performance task

LDX reduced commission errors (*t*(19) = −2.11, *p* = 0.048, *d* = −0.47) on non-target trials and reduced SDRT/RTV (*t*(19) = −2.23, *p* = 0.04, *d* = −0.50) relative to placebo. LDX had no effect on target (non-X trials) omission errors (*t*(18) = −0.52, *p* = 0.61, *d* = −0.12) nor target RT (*t*(19) = 1.46, *p* = 0.16, *d* = 0.33) (Table 1).

#### Emotional test battery and n-back working memory task

The only statistically reliable effect of LDX versus placebo was to reduce RT in the emotional categorisation task (*F*(1, 20) = 10.42, *p* < 0.01, η_p_^2^ = 0.34). There were no effects of LDX on working memory performance in the n-back task (Supplementary Tables 3, 4 and 5).

### fMRI picture rating task

#### Behavioural

There was an interaction between drug condition and stimulus type (fat and sugar content of stimulus foods) *F*(3) = 4.76, *p* = 0.005, η_p_^2^ = 0.185. Follow-up post-hoc tests revealed lower ratings of high-fat, low sugar foods after LDX (mean = 3.73) versus placebo (mean = 4.07), *t*(20) = 2.61, *p* = 0.009, *d* = −0.65.

#### BOLD responses

*Food versus Non-food:* Statistically significantly greater (whole-brain FWE-corrected) BOLD responses to food compared to non-food images were observed in the large bilateral distributed network. It included clusters in the bilateral insula, bilateral mid frontal, left precuneus, left orbitofrontal cortex, left middle occipital gyrus, left superior frontal, left thalamus, left midcingulate, left precentral, left angular gyrus, right superior frontal gyrus. (Supplementary Table 6).

**Fig. 4:**
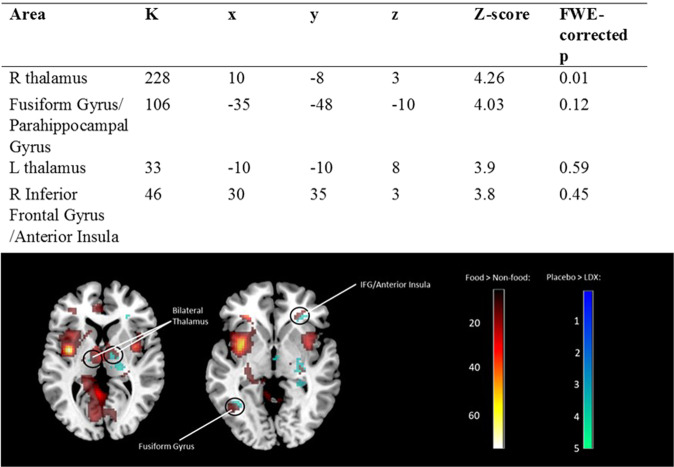
Clusters significant for contrast Placebo > LDX at initial uncorrected detection threshold *p* < 0.001. L = left, R = right (top panel). Regions showing greater responses to food (red) compared to non-food overlaid with regions showing greater responses to food on placebo compared to LDX (blue). Image clusters thresholded at *p* < 0.001 uncorrected (bottom panel).

*Interaction of LDX effect and food:* Fig. 4 shows the regions where activity was attenuated by LDX only for food stimuli (cyan -green) and the areas showing the main effect of food > non-food (red-orange). Under a whole-brain FWE-corrected significance peak threshold, the only contrast that remained significant was for the right thalamus. Left thalamus was significant (*#voxels* = 26, *Z* = *3.8*, small volume FWE-corrected −6 mm sphere around the food > no food peak *p* = 0.001, *d* = 0.85). There were no other above threshold specific food responses to LDX manipulation within the food > non-food network.

*Psycho-physiological Interaction Analysis:* Effective functional connectivity (for food > non-food) between the left thalamus and left middle insula was attenuated in the LDX condition relative to placebo (a trend effect: *#voxels* = 9, *Z* = 2.17, small volume corrected with a 4 mm sphere at [−37, 5, 7], *p* = 0.069, *d* = 0.50). There were no above threshold changes to functional connectivity of the right thalamus by LDX.

## Discussion

This study showed that a single dose of LDX was associated with reduced intake of pasta and cookies in women with binge eating symptomatology compared to placebo. Participants ate fewer grams per minute of pasta in the LDX condition than in the placebo condition, and ratings of palatability for pasta were significantly reduced in the LDX condition at the end of the meal. Appetite ratings were reduced after LDX, and ratings of arousal and physical effects (lightheaded, nausea, and faint) were increased. LDX did not affect performance on the n-back task but did improve sustained attention and reduced responding on some but not all measures of impulsivity.

The finding that LDX reduced consumption of both the pasta meal and the cookie snack but had differential effects on the microstructure of eating the two foods suggests that it may be acting via at least two neural mechanisms. LDX reduced the eating rate of pasta and reduced rated palatability at the end of the meal but had no effect on initial palatability ratings. These actions are signatures of satiety enhancing manipulations, e.g. administration of serotonin 5-HT_2C_ receptor agonists [15]. As LDX increases serotonin transmission, as well as dopamine and noradrenaline transmission [33], enhancement of serotonergic satiety mechanisms is consistent with the known pharmacological effects of the drug. On the other hand, the finding that LDX also reduced cookie intake without affecting eating rate suggests that the drug reduces motivation to eat when satiated possibly via a state-dependent reduction in the reward value of food [34]. A review and meta-analysis of the effects of LDX on feeding in rodents have similarly found that the drug reduces both standard lab chow and palatable food intake in binge eating models [7–9, 35]. Taken together, these data suggest that LDX may decrease binge eating through a dual action of reduced motivation to consume highly palatable binge foods and enhancement of satiety.

The effects of LDX on food consumption may also be explained by a drug-induced reduction in aspects of impulsivity/improvement in attention, both of which have been linked to the control of food intake [36]. LDX improved sustained attention and reduced commission errors that are indicative of impulsive responding in the Continuous Performance Task. LDX had no statistically significant effect on SST performance but there was a trend toward reduced impulsive responding to prepotent stimuli as reflected by reduced commission errors. These findings are in keeping with LDX-induced reductions in impulsivity reported in preclinical models of binge eating [36]. Clinical reports of the effects of LDX on impulsivity in BED patients are limited to a single clinical trial in which a reduction was reported as trait impulsivity using the BIS [4, 37]. Moreover, there has been no investigation to date on the effects of LDX on sustained attention in BED. Hence, for the first time, we demonstrate an effect of LDX to improve sustained attention and reduce task-based impulsive responding in the context of a continuous performance task in participants with binge eating symptoms. LDX has been reported to improve working memory performance in non-binge eating participants [38, 39], but the absence of an effect of LDX on performance on a working memory task does not support the suggestion that LDX brings about its therapeutic effects via improvements in working memory.

fMRI analysis revealed that LDX reduced activity bilaterally in the thalamus and tended to reduce functional connectivity between the thalamus and the insula but had no significant or trend significant effects on other brain areas classically involved in reward and cognition. The only other study to examine the effects of LDX on BOLD responses was suggestive of lower thalamic activation after 12 weeks of LDX treatment as compared to baseline in patients with BED, although this effect was not significant likely due to the small sample size and there was no placebo condition. However, at baseline, binge eating scores significantly correlated with activation in the thalamus and changes in thalamus activation were positively correlated with changes in binge eating scores following LDX treatment. Taken together, these data suggest that further investigation of the role of the thalamus in mediating the therapeutic effect of LDX, specifically in relation to food are warranted. The thalamus receives extensive dopaminergic input and plays a role in the processing of incentive sensory information [40], including driving attention to motivationally salient external cues [41]. The thalamus conveys information about the motivational salience of exteroceptive stimuli to the insula, which integrates this information with interoceptive information about bodily state to direct motivational behaviour [42]. A possible explanation for the effects of LDX in the present study is that when women with binge eating view food pictures, the thalamus is strongly activated, which is reflected in heightened responsiveness to food cues. LDX reduces this activation and the functional connectivity with the insula, a likely outcome of which is a reduction of the relative influence of exteroceptive versus interoceptive signals in response to food. Such an interpretation is consistent with the observed effects of LDX to reduce liking for food when satiated. Future studies could test this hypothesis by examining the neural responses to LDX using tasks that involve the integration of exteroceptive and interoceptive cues e.g. sensory specific satiety.

It is unlikely that the reduction of food intake by LDX is secondary to adverse effects of the drug on mood and physical state. On measures of self-reported mood, LDX increased ratings of arousal and physical effects (lightheaded, nausea, and faint), which is consistent with previous reports from clinical studies [43]. However, the relatively low reporting levels both here and in RCTs indicates that physical effects such as nausea are not a primary cause for the reduction in food intake observed in this study, especially because palatability ratings were only reduced at the start and not the end of the meal. In addition, the lack of effect of LDX on ETB measures suggests that the drug did not induce changes in mood that could account for its reductions in food intake. Although increases in blood pressure and heart rate that may accompany arousal have been found to affect overall cerebral blood flow [44], such an effect is unlikely to explain the reduction in neural activity induced by LDX. Importantly, the effect of LDX reported is on the contrast food > non-food and as such, the subject-level variable carried forward into group level analysis controls for any global effects on variables such as cerebral blood flow. This is because these effects would be the same for both the food and non-food stimuli and so would not feature in the analysis of the drug effect on the food > non-food contrast. The present study was not designed to assess whether the effects of LDX are dependent upon the severity of binge-eating symptoms, but this could be examined in future studies by testing whether greater effects of the drug are observed for participants with more severe symptoms.

In summary, we have documented for the first time the behavioural and neural profile of the effects of an acute dose of 50 mg LDX in women with binge-eating symptomatology. We find that LDX has multiple effects to enhance satiety and reduce food-reward related responding and to improve cognitive control. Our fMRI results highlight a potential role of the thalamus in mediating the action of LDX to reduce appetite via changes in the balance of exteroceptive versus interoceptive control. These data provide novel mechanistic insights into LDX in the context of binge eating and suggest that novel drugs to treat BED might be most effective if they combine effects on appetite/satiety, reward, and cognitive processes.

## Supplementary information

Supplemental

## Data Availability

Data for this study will be made available in a public archive following publication of this study. In the interim, data are available upon request.
