# Supplementary material for: The effects of lisdexamfetamine dimesylate on eating behaviour and homeostatic, reward and cognitive processes in women with binge-eating symptoms: an experimental medicine study
Source: Transl Psychiatry. 2022 Jan 10;12:9. doi: 10.1038/s41398-021-01770-4 (PMC8744047; doi:10.1038/s41398-021-01770-4)
Supplement: Supplementary file 1 — Supplemental [file 41398_2021_1770_MOESM1_ESM.docx]

**Supplemental materials/tables**

**fMRI methods**

**Image Acquisition**

Images were acquired using a Siemens MAGNETOM Prisma 3T MRI system at the Centre for Human Brain Health, University of Birmingham using a 64-channel head coil. Functional images were acquired during the task (3 runs x 300 volumes) using an echo-planar imaging (EPI) sequence with the following parameters: repetition time (TR = 1500 ms), multiband accelerator factor = 3, flip angle =71°, echo time (TE = 35 ms), and 57 slices (voxel size = 2.5 × 2.5 × 2.5 mm^3^). Structural T1-weighted images were acquired with the following parameters: repetition time (TR = 2000 ms), flip angle = 8° 208 slices (voxel size = 1 x 1 x 1 mm). Additionally a field map was acquired with the following parameters: echo time (short = 4.92 ms, long = 7.38 ms), slice thickness = 3mm.

**Pre-processing**

All pre-processing and statistical analysis was conducted using SPM12 (Wellcome Department of Imaging Neuroscience, London, UK) run with MATLAB 2019 (Mathworks Inc, Natick, MA). Pre-processing steps included: slice-timing correction, functional image realignment, calculation of a voxel displacement map using the fieldmap which was used to unwarp functional images, and coregistration of the T1 anatomical image to the mean functional image. The T1-weighted images were then segmented into white and grey matter and this was inputted into the DARTEL toolbox to create a group template, which was normalised to MNI space. Images were smoothed using a Gaussian kernel of 8 mm and resliced with a voxel size 2.5 x 2.5 x 2.5 mm. Task runs that included head movement >3mm were excluded from further analysis. One participant was excluded from analysis due to excessive movement in all task runs. Eight participants had one task run excluded from analysis (3 LDX runs, 5 placebo). Two participants had two task runs removed (all placebo).

**General Linear Model Analysis**

For each subject a model was defined containing 8 regressors: four food stimulus categories (high fat, high sugar; high fat, low sugar; low fat, high sugar and low fat, low sugar) and four matched non-food stimulus categories. Six motion parameters were also included. The response to events was modelled by a canonical hemodynamic response function. This resulted into four different contrast images: food items (four food categories) vs. matched non-food items. These contrast images were entered into a second-level two-way ANOVA with condition (drug and placebo) and food type (high fat high/low sugar, low fat high/low sugar).

Main effects and interactions were considered significant at p < 0.05 family-wise error (FWE) correction using a primary voxel-level uncorrected threshold of p < 0.001. Small-volume correction was applied to a priori defined regions of interest (insula, ventral striatum, lateral orbitofrontal cortex, dorsolateral prefrontal cortex, parahippocampal gyrus, ventromedial prefrontal cortex, anterior cingulate cortex, dorsal striatum, thalamus and globus pallidus). Coordinates were based on peaks taken from the food > non-food contrast and peaks from responses to food cues in existing literature [45, 46, 47, 48, 48].

**Psycho-physiological Interaction (PPI)**

Regions (if any) showing a main effect of drug were used as seed regions for PPI analysis to assess connectivity changes associated with LDX administration. 4mm spheres around the peak of these areas were defined and the first eigenvariate for this volume of interest extracted. PPI analysis was run to determine the interaction between the time-series and food/non-food stimuli and results of this model was entered as a regressor into a group level GLM with one factor (drug and placebo). Small volume correction was applied to regions determined to be involved in processing of food rewards (insula coordinates for correction [-37, 5, 7] obtained from food > non-food contrast and meta-analysis of BOLD response to food cues [46]).

Supplemental Table 1. Inclusion/exclusion criteria

| Inclusion Criteria: | Exclusion Criteria: |
| --- | --- |
| 1. Aged 18-55 2. Female 3. Fluent English speaking 4. Minimum body mass index (BMI) of 18.5 5. Minimum score of 18 on the Binge Eating Scale 6. Medical clearance including ECG screening 7. Liking of study foods 8. No special diets (e.g., vegan or vegetarian) | 1. Current diagnosis or symptoms of Bulimia Nervosa or Anorexia Nervosa 2. Psychotherapy or psychopharmacological treatment for Binge Eating Disorder in the last 3 months 3. Metabolic disorder 4. Current mental health (excluding Binge Eating Disorder) disorder determined via the SCID or self-reported diagnosis. 5. Substance use disorder 6. Current smoker 7. Pregnant or breastfeeding 8. Positive breathalyser on the morning of testing 9. Food allergies related to the study 10. MRI-related exclusion 11. Weight exceeding 152 kg 12. Left-handed 13. Limited or increased perception of temperature changes 14. Pathological hearing 15. Surgical operation less than 3 months ago 16. Moderate or severe head injury 17. Acute illness or infection in the last month 18. Claustrophobia 19. Non-removable metal |

*Note: 8 participants were screened out due to exclusion criteria*

Supplemental Table 2. Participant characteristics

| Characteristic (*N*=22) | *M* (±SE) | Min and Max Score |
| --- | --- | --- |
| Age | 24.41 (1.77) | 18-49 |
| BMI | 26.35 (1.22) | 19.5-41 |
| BES (0-46) | 28.36 (1.63) | 18-40 |
| DEBQ (1-5) |  |  |
| *Restraint* | 2.99 (0.14) | 1.6-3.7 |
| *External Eating* | 3.92 (0.11) | 2.8-4.6 |
| *Emotional Eating* | 3.52 (0.17) | 2-4.6 |
| BDI (0-63) | 11.80 (1.55) | 1-26 |
| BIS |  |  |
| *Total (30-120)* | 68.17 (2.47) | 44-86 |
| *Attention (8-32)* | 17.70 (1.06) | 10-26 |
| *Motor (11-44)* | 24.55 (1.09) | 15-36 |
| *Non-Planning (11-44)* | 26.45 (0.96) | 15-32 |

Mean ± standard error of the data presented alongside each measure’s minimum and maximum score obtained. BMI: Body Mass Index; BES: Binge Eating Scale; DEBQ: Dutch Eating Behaviour Questionnaire; BDI: Beck Depression Inventory; BIS: Barratt Impulsiveness Scale

Supplemental Table 3. Emotional Test Battery results

|  | |  | Negative Valence | | | Positive Valence | |
| --- | --- | --- | --- | --- | --- | --- | --- |
| ETB Task | **Measure** | | | **Placebo** | **LDX** | **Placebo** | **LDX** |
| Emotional Categorisation (ECAT) | Accuracy (%) | | | 96.82 (1.02) | 95.45 (1.71) | 94.32 (1.20) | 94.55 (1.23) |
|  | Reaction time (ms) | | | 1476.38 (59.30) | 1317.43 (34.43) | 1353.95 (45.20) | 1239.52 (32.48) |
| Emotional Recall (EREC) | Correct words | | | 3.45 (0.41) | 3.91 (0.38) | 6.68 (0.68) | 6.10 (0.51) |
|  | Commission | | | 1.23 (0.34) | 1.41 (0.38) | 2.50 (0.45) | 2.23 (0.47) |
| Emotional Recognition Memory (EMEM) | Accuracy (%) | | | 80.44 (2.00) | 83.09 (0.92) | 83.24 (1.78) | 86.03 (1.66) |
|  | Commission (%) | | | 18.89 (2.00) | 17.64 (1.13) | 16.39 (1.72) | 14.72 (1.74) |
|  | Reaction time (ms) | | | 1401.18 (69.22) | 1442.10 (72.30) | 1401.27 (71.82) | 1372.73 (66.37) |

Mean ± standard error presented for Emotional Test Battery (ETB). LDX reduced (*p* < 0.05) reaction time for negatively and positively valenced words in the ECAT compared to placebo.

Supplemental Table 4. Facial Expression Recognition Task results

|  | Accuracy (%) | | | Commission Errors (%) | | Reaction Time (ms) | |
| --- | --- | --- | --- | --- | --- | --- | --- |
| Valence | | **Placebo** | **LDX** | **Placebo** | **LDX** | **Placebo** | **LDX** |
| Neutral | | 87.57 (1.64) | 81.12 (2.53) | 27.44 (1.00) | 27.90 (1.27) | 1545.48 (50.68) | 1622.27 (74.40) |
| Sad | | 67.26 (2.19) | 71.69 (1.96) | 3.89 (0.49) | 3.93 (0.75) | 1471.17 (31.16) | 1565.48 (39.01) |
| Anger | | 61.20 (2.43) | 63.31 (2.43) | 1.92 (0.37) | 2.02 (0.50) | 1828.00 (58.76) | 1876.18 (60.10) |
| Disgust | | 70.00 (2.48) | 70.28 (1.68) | 1.54 (0.36) | 1.61 (0.27) | 1797.05 (74.67) | 1781.15 (65.04) |
| Fear | | 52.89 (3.23) | 50.87 (3.60) | 1.20 (0.21) | 1.07 (0.20) | 2103.84 (75.30) | 2231.83 (88.73) |
| Surprise | | 69.08 (1.49) | 68.99 (1.71) | 3.09 (0.70) | 3.53 (0.67) | 1621.35 (54.16) | 1738.98 (95.27) |
| Happy | | 54.18 (2.40) | 49.47 (2.12) | 0.86 (0.20) | 0.77 (0.16) | 1607.28 (34.60) | 1688.25 (51.13) |

Mean ± standard error presented for Facial Expression Recognition Task (FERT).

Supplemental Table 5. N-back results

|  | Accuracy (%) | | Reaction time (ms) | |
| --- | --- | --- | --- | --- |
| Stimuli | **Placebo** | **LDX** | **Placebo** | **LDX** |
| 2-back | 83.88 (2.71) | 84.63 (2.56) | 712.55 (46.85) | 689.83 (44.48) |
| 3-back | 76.63 (3.49) | 74.25 (3.17) | 726.16 (52.49) | 725.55 (50.73) |

Mean ± standard error presented for N-back.

Supplemental Table 6. Clusters significant for contrast Food > Non-food at FWE-corrected p = 0.01. L = left, R = right.

| **Area** | **K** | **X** | **Y** | **Z** | **Z-score** | **FWE-corrected p** |
| --- | --- | --- | --- | --- | --- | --- |
| Insula (L) | 349 | -37.5 | -5 | 7.5 | 7.68 | <0.001 |
| Midfrontal (L) | 117 | -35 | 35 | 20 | 7.26 | <0.001 |
| Precuneus (L) | 348 | -5 | -50 | 17.5 | 6.94 | <0.001 |
| Inferior Orbitofrontal (L) | 10 | -30 | 32.5 | -5 | 6.15 | 0.008 |
| Inferior Temporal (L) | 55 | -47.5 | -47.5 | -20 | 6.11 | <0.001 |
| Mid Occipital (L) | 89 | -25 | -65 | 37.5 | 6.1 | <0.001 |
| Insula (R) | 97 | 37.5 | 0 | 7.5 | 5.95 | <0.001 |
| Superior Frontal (L) | 322 | -15 | 30 | 52.5 | 5.94 | <0.001 |
| Thalamus (L) | 33 | -2.5 | -10 | 7.5 | 5.33 | <0.001 |
| Mid Cingulate (L) | 27 | -2.5 | 0 | 32.5 | 5.29 | 0.002 |
| Precentral (L) | 46 | -47.5 | 10 | 35 | 5.27 | <0.001 |
| Angular Gyrus (L) | 13 | -45 | -65 | 37.5 | 4.91 | 0.006 |
| Superior Frontal (R) | 10 | 17.5 | 40 | 42.5 | 4.89 | 0.009 |
| Midfrontal (R) | 11 | 37.5 | 37.5 | 17.5 | 4.8 | 0.008 |

References

45. Thomas JM, Dourish CT, Tomlinson J, Hassan-Smith Z, Hansen PC, Higgs S. The 5-HT2C receptor agonist meta-chlorophenylpiperazine (mCPP) reduces palatable food consumption and BOLD fMRI responses to food images in healthy female volunteers. *Psychopharmacology (Berl)*. 2018;235(1):257-267. doi:10.1007/s00213-017-4764-9

46. Tang DW, Fellows LK, Small DM, Dagher A. Food and drug cues activate similar brain regions: a meta-analysis of functional MRI studies. *Physiol Behav*. 2012;106(3):317-324.

47. Pelchat ML, Johnson A, Chan R, Valdez J, Ragland JD. Images of desire: food-craving activation during fMRI. *Neuroimage*. 2004;23(4):1486-1493.

48. Chen EY, Zeffiro TA. Hunger and BMI modulate neural responses to sweet stimuli: fMRI meta-analysis. *Int J Obes (Lond)*. 2020;44(8):1636-1652.

49. Brooks SJ, Cedernaes J, Schiöth HB. Increased Prefrontal and Parahippocampal Activation with Reduced Dorsolateral Prefrontal and Insular Cortex Activation to Food Images in Obesity: A Meta-Analysis of fMRI Studies. *PLoS One*. 2013;8(4):e60393. doi:10.1371/journal.pone.0060393
